# Asymptomatic Pulmonic Valve Mass: A Diagnostic and Therapeutic Dilemma

**DOI:** 10.7759/cureus.35104

**Published:** 2023-02-17

**Authors:** Wahab J Khan, Muhammad Asif, Ifrah Nadeem, Mashood B Badshah

**Affiliations:** 1 Internal Medicine, University of South Dakota Sanford School of Medicine, Sioux Falls, USA

**Keywords:** right ventricular cardiac mass, cardiac mass, cardiac mass tumor, dual antiplatelet therapy, pulmonic valve, medical management, myxoma

## Abstract

Cardiac masses are not common but remain important in cardiology practice as they can cause havoc to a patient's life through obstructive and arrhythmogenic symptoms. These lesions mostly include vegetation, thrombi, and tumors. Myxomas are the most common primary cardiac tumor, primarily arising from the left heart chambers. It is exceedingly rare for a myxoma to emerge from the right-sided cardiac valves. The standard treatment is surgical resection, regardless of size, which is not always possible. We report a unique case of a male with multiple co-morbidities who presented with an incidental finding of a pulmonary valve mass suspicious of being a myxoma. The myxomatous mass was asymptomatic with no right ventricular outflow tract obstruction. Echocardiogram can help identify and characterize these lesions, but this may not be easy, especially in the case of atypical location or morphology of the mass. Similarly, in some cases, the patient may not be able to undergo surgical excision. In such cases, there is no consensus or guidelines to help clinicians best manage the patients medically, creating a diagnostic and therapeutic dilemma.

## Introduction

Cardiac masses are not uncommon. These can be divided into neoplastic and non-neoplastic. The non-neoplastic category includes mainly vegetations and thrombi. The neoplastic group is classified into two subgroups: 1) primary cardiac tumors (PCT), which could be benign or malignant, and 2) secondary cardiac tumors, which by definition are malignant. In the heart, metastatic or secondary tumors are more common than PCT [1]. Among PCT, cardiac myxomas (CM) are the most common. They are most commonly located in the left atrium; however, in 15-20% of the cases, they may occur in the right atrium [2]. Histologically, these tumors comprise dissipated cells inside a mucopolysaccharide stroma. CM produces growth factors, including vascular endothelial growth factor, presumably augmenting angiogenesis and beginning the initial phases of cancer development [3]. CM is generally pedunculated and thick in consistency; the surface may be smooth, villous, or friable [4]. The cardiovascular signs rely on the anatomic site of the tumor. CM arising from the valves is a rare phenomenon. Herein we report a case of a 77-year-old patient with a cardiac mass representing PCT suspicious for myxoma emerging from the pulmonary valve.

## Case presentation

A 77-year-old Caucasian male presented to the emergency department with worsening shortness of breath, night sweats, low-grade fever, and aggravating fatigue for four months. He denied chest pain, cough, orthopnea, and paroxysmal nocturnal dyspnea. His medical history was significant for chronic kidney disease stage 3 (CKD3), type 1 diabetes mellitus (DM1) complicated by neuropathy, uncontrolled hypertension, and peripheral vascular disease. On admission, his vital signs included a blood pressure of 160/90 mmHg, a temperature of 98.6 °F, a heart rate of 78 beats/min, and oxygen saturation (SaO2) of 90% on room air. On physical examination, the cardiac examination was unremarkable; however, the patient had significant bilateral pedal edema up to the knee. Initial chest x-ray was concerning for bilateral pleural effusions and pulmonary vascular congestion. The electrocardiogram showed left ventricular hypertrophy with secondary repolarization abnormalities, multiple premature ventricular contractions, and ST-segment depressions in V4-V6. Pertinent lab findings included a brain natriuretic peptide level of 2932 pg/mL (0-100pg/ml) and troponin I of 0.458 ng/mL (<0.013ng/ml) with a flat trend. Serum creatinine was 1.72, and Hb was 15.0, both of which were around his baseline. The patient was admitted for non-ST elevation myocardial infarction and acute new onset decompensated heart failure. Transthoracic echocardiogram (TTE) showed a left ventricular ejection fraction of 20-25% with global hypokinesis and a 1.5 x 1.7 cm mass in the right ventricular outflow tract (RVOT).

**Figure 1:**
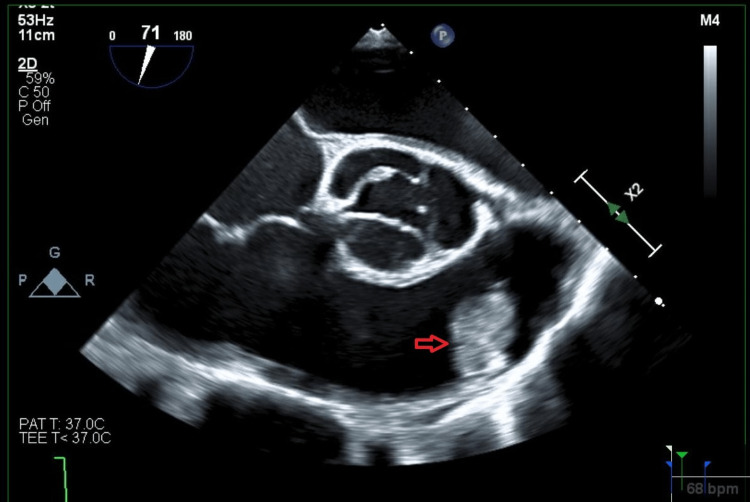
Mid-esophageal right ventricle inflow and outflow view on transesophageal echocardiogram. Suspected myxoma (red arrow) seen on the pulmonic valve leaflet.

**Figure 2:**
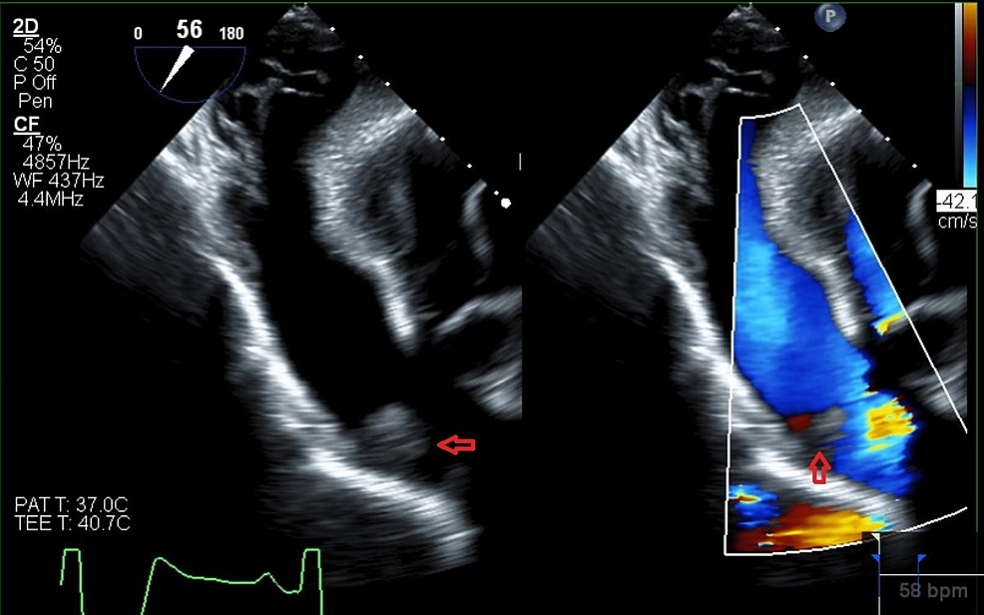
Transgastric right ventricle outflow view on transesophageal echocardiogram. No significant systolic flow acceleration on the color doppler (left panel). Suspected myxoma on the pulmonic valve leaflet (red arrows)

Further evaluation with transesophageal echocardiogram (TEE) revealed the mass to be a homogenous avascular echo-density 1.5 x 1.7 cm on the ventricular side of the anterior pulmonic valve leaflet suspicious of CM. The mass's motion depended on the pulmonic valve (Figure 1, Video 1). There was no pulmonic valve stenosis (Figure 2), and only mild pulmonary regurgitation was evident. Left heart catheterization showed severe three-vessel disease with complete right coronary artery occlusion along with 95% blocked mid-left anterior descending artery and 90% distal circumflex artery occlusion.

**Video 1:** Mid-esophageal right ventricle inflow and outflow view

In the absence of echocardiographic RVOT obstruction or pulmonic valve stenosis along with normal right ventricular systolic function and right ventricular systolic pressure of 25mmHG, the patient's acute symptoms were thought to be secondary to ischemic cardiomyopathy (ICM) with depressed ejection fraction leading to acute congestive heart failure (CHF). Unfortunately, a cardiac CT or MRI could not be performed due to poor renal reserve.

**Figure 3:**
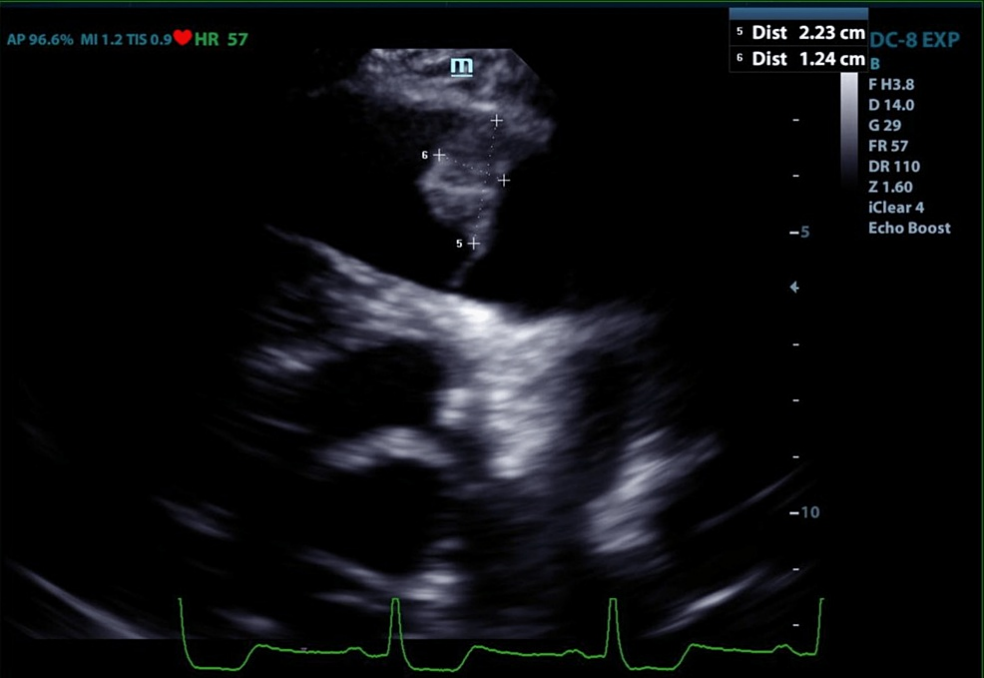
Pulmonic valve mass on follow-up transthoracic echocardiogram (parasternal short axis right ventricle outflow view)

Due to advanced age and multiple co-morbidities, the patient was a poor surgical candidate for open-heart surgery, especially with the attempted tumor resection likely requiring valve replacement. He subsequently underwent high-risk staged percutaneous coronary intervention (PCI) followed by dual antiplatelet therapy (DAPT) with conservative management of CM as an outpatient. The patient had no stroke, pulmonary embolism or hospital admissions, or any other complications from myxoma at six months follow-up. In addition, his constitutional symptoms have improved. Repeat limited echocardiogram at six months showed an improvement in ejection fraction (EF) from 25% to 40-45% and stable pulmonic mass at the same location (Figure 3).

## Discussion

Primary cardiac tumors are rare. Most tumors are benign, and more than half of those benign tumors are myxomas. Most of them involve the left side, but the involvement of the right side is not uncommon. Valves are rarely affected, and the involvement of pulmonic valves is highly unusual [5,6]. Sometimes, CM can originate from multiple cardiac chambers simultaneously and be a part of the Carney complex (a combination of atrial and extracardiac myxomas, schwannomas, and various endocrine tumors along with skin pigmentation issues).

CM affects the middle-aged population, with a female predilection of approximately 3:1 [5]. Right-sided CM is rarely reported in the elderly [7,8]. It may present as valvular obstruction or stenosis, causing syncope and other cardiovascular symptoms. On the other hand, it could present with thromboembolic phenomena like strokes and pulmonary emboli. In the end, most myxomas also have constitutional symptoms of fever, fatigue, and chills. A few may be asymptomatic and found incidentally. Diagnosis can almost always be made by echocardiography. TTE and TEE can show a tumor and its relation to the atrial septum. Typical appearances on TTE include homogenous or heterogeneous tumors, sometimes with calcification in about 10% of cases. In the absence of distinctive characteristics on echocardiography, cardiac CT or MRI may be helpful. Myxomas appear as low-attenuating intracavitary masses, sometimes with calcifications. On MRI, a myxoma may manifest as a heterogeneous mass on T1- and T2-weighted images. However, at times advanced imaging may not be available, or the risks associated with these modalities may limit their use.

The most critical differentials to consider are a thrombus and valvular vegetations, both of which usually occur in severe atrial dilation, central catheters, atrial fibrillation, and pacemaker [9]. Occasionally, however, surgery is required to confirm the diagnosis. Alternatively, a short course of anticoagulation may help differentiate between the two if the mass is small. Sometimes it is also challenging to exclude myxoma from fibroelastoma without definitive histologic analysis. Certain morphologic features that favor the diagnosis of myxoma, as in our case, include the larger size and origin from the base of the leaflet instead of from the tip of the leaflets. Fibroelastomas usually have leaflet-independent motion and occur downstream [10,11]. Other differentials include secondary cardiac tumors, hemangioma, granuloma, lymphoma, and infectious/inflammatory etiologies like tuberculosis and vasculitis.

CM produce vascular endothelial growth factor (VEGF) and platelet-derived growth factor (PDGF), among other cytokines that are responsible for constitutional symptoms [12]. In addition, it is also well known that aspirin and clopidogrel reduce PDGF and VEGF expression [13,14]. Therefore, theoretically, these antiplatelet agents may have a role in protecting myxoma patients against thromboembolic and constitutional symptoms.

The standard treatment of CM is surgical resection. With increasing life expectancy, however, the incidence of myxomas is rising in the elderly, creating challenges for surgical treatment [8,15]. A recent study reported about 25% mortality in the geriatric population undergoing cardiac surgery with a mean age of ~69±4 years at the time of surgery [16]. There have been reports of no surgical intervention, but the literature is silent about non-surgical management, follow-up, and prognosis in these patients. The role of anticoagulation for patients with CM is not established since there is no evidence that it can prevent embolic events in these patients [17]. A recent study showed that out of 265 patients, the mortality rate amongst 64 who did not undergo surgery was 17.2% (11 patients) during the follow-up for 24.6±36.5 months. It was unclear whether they were treated with anticoagulation or antiplatelets [18]. A recent case report described a patient who survived three months on apixaban without any complications [15].

However, further work is required to evaluate potential treatments for CM in patients who are poor surgical candidates for resection. Our patient has been on DAPT for six months with no complications, including strokes, pulmonary emboli, or worsening dyspnea. Repeat echo shows the stable mass at the same location (Figure 3). Unfortunately, further characterization to confirm myxoma via a cardiac CT or MRI could not be performed in our patient due to concerns for poor renal reserve and the need for contrast.

## Conclusions

CM is the most common benign tumor of the heart. It can involve valvular surfaces, although not common. The standard treatment is surgical resection. However, it can sometimes be challenging to confirm the diagnosis and treat it definitively due to the patient's poor renal or cardiac reserve to undergo advanced imaging and surgical excision, respectively.

Antiplatelet agents may help reduce both thromboembolic and constitutional symptoms in patients who are poor surgical candidates for resection by decreasing the expression of agents like PDGF and VEGF via antiplatelet activity. However, more studies and follow up of patients not undergoing surgical management are needed to devise the best medical strategy to help these patients avoid thromboembolic complications.
